# Viral RNAs Are Unusually Compact

**DOI:** 10.1371/journal.pone.0105875

**Published:** 2014-09-04

**Authors:** Ajaykumar Gopal, Defne E. Egecioglu, Aron M. Yoffe, Avinoam Ben-Shaul, Ayala L. N. Rao, Charles M. Knobler, William M. Gelbart

**Affiliations:** 1 Department of Chemistry & Biochemistry, University of California Los Angeles, Los Angeles, California, United States of America; 2 Institute of Chemistry & The Fritz Haber Research Center, The Hebrew University of Jerusalem, Givat Ram, Jerusalem, Israel; 3 Department of Plant Pathology, University of California Riverside, Riverside, California, United States of America; Wuhan University, China

## Abstract

A majority of viruses are composed of long single-stranded genomic RNA molecules encapsulated by protein shells with diameters of just a few tens of nanometers. We examine the extent to which these viral RNAs have evolved to be physically compact molecules to facilitate encapsulation. Measurements of equal-length viral, non-viral, coding and non-coding RNAs show viral RNAs to have among the smallest sizes in solution, i.e., the highest gel-electrophoretic mobilities and the smallest hydrodynamic radii. Using graph-theoretical analyses we demonstrate that their sizes correlate with the compactness of branching patterns in predicted secondary structure ensembles. The density of branching is determined by the number and relative positions of 3-helix junctions, and is highly sensitive to the presence of rare higher-order junctions with 4 or more helices. Compact branching arises from a preponderance of base pairing between nucleotides close to each other in the primary sequence. The density of branching represents a degree of freedom optimized by viral RNA genomes in response to the evolutionary pressure to be packaged reliably. Several families of viruses are analyzed to delineate the effects of capsid geometry, size and charge stabilization on the selective pressure for RNA compactness. Compact branching has important implications for RNA folding and viral assembly.

## Introduction

Single-stranded (ss) RNA molecules are typically branched, with physical properties that depend on the secondary and tertiary structures determined by their primary nucleotide (nt) sequence [1]–[3]. High-resolution structures have been elucidated for several biologically important molecules with lengths up to hundreds of nt; e.g. ribozymes, transfer RNAs, and messenger RNA sub-sequences [4]–[6]. For longer sequences, however, it is generally not possible to identify a unique secondary/tertiary structure that dominates the ensemble of configurational states of the molecule [7], [8]. [An important exception is that of ribosomal RNAs [9], but there the structures of these thousands-of-nt-long RNA molecules are largely determined by the many proteins with which they are bound.] Coarse-grained statistical properties – such as radius of gyration and shape anisotropy – have been measured for viral RNAs several thousand nt long [8], [10], but how these relate to the primary sequence or even the underlying secondary structures has not been studied systematically.

On the other hand, the statistics of model branched molecules and aggregates are well studied [11]–[13], e.g. “star” polymers, dendrimers, diffusion-limited-aggregation clusters, mathematical tree structures and ideal randomly-branched polymers. In each case, it is possible to predict and/or measure the radius of gyration as a function of molecular weight (number of monomeric units). Very few experiments and theories [7], [14]–[19] extend this approach to long RNA molecules. In particular, the connection between primary sequence and branching properties, and the resulting molecular sizes, has not been studied in long RNAs.

ssRNA viral genomes are special RNA molecules in several significant ways. First, because they involve two or more genes, they are necessarily thousands of nt long. Also, unlike other long RNAs, such as edited messenger RNA transcripts or ribosomal RNA, they are constrained to have physical sizes compatible with being packaged spontaneously by viral coat proteins into small volumes corresponding to the inside of a rigid viral capsid. As proposed in earlier theoretical work [7], [14], the above factors suggest that viral RNA molecules might be more compact than other sequences of identical length, in order to enhance their encapsulation efficiency and hence the survivability of the virus.

In this paper, we study the correlation between the nt sequence and the physical size of large RNA molecules – in particular, whether the sequence of a viral RNA codes not just for required protein products but also for its own physical size. We compare the experimentally determined size of a 2117-nt viral RNA with those of non-viral sequences of identical length and find the viral sequence to be the most compact. The relative sizes of these sequences are explained by analyzing the nature of branching in predicted ensembles of secondary structures. We show that compactness originates from an increased density of branching defined by the number, degree and organization of multi-helix junctions in secondary structures. We compare several families of ssRNA viruses and find that genomes with propensity for denser branching typically belong to families where other means of RNA compaction (e.g., polyvalent cations) may not exist. Finally, we outline how compactness improves the robustness of RNA folding and enhances the ability of a viral genome to package in a capsid.

## Results

### RNA Sequence and Buffer Choice

To test the relationship between the primary nt sequence of an RNA and its physical size, we study nine RNAs of identical lengths (2117 nt), but different nt compositions and biological functions. The first is genomic RNA3 of Brome Mosaic Virus (BMV) [20], a plant pathogen. BMV RNA3 (B3) is a two-gene plus-strand RNA coding for a movement protein (MP) and a capsid protein (CP). The second molecule is the anti-sense (i.e. reverse-complement or minus-strand) RNA of B3, hereafter denoted as B3A (BMV RNA3 Anti-sense). An anti-sense strand can differ in composition and pattern only in the unpaired regions of the sense strand, therefore representing a sequence with most of the original nt patterns and about 20% change in composition. The third molecule is a B3 mutant, hereafter called B3R (BMV RNA3-Reverse), with the positions of the MP and CP genes swapped. This alteration changes the overall sequence, but not the nt composition. It also hampers the ability of B3 to package into virions both *in vivo* and *in vitro* [20]. The fourth molecule is the anti-sense strand of B3R, denoted as B3RA (BMV RNA3 Reverse Anti-sense).

To compare the four B3-based RNAs with those not expected to have evolved with a selective pressure to be compact, the remaining five RNAs were transcribed from arbitrarily chosen non-overlapping sections of the yeast genome (see Methods). Labeled Y1 through Y5, three (Y1, Y2 and Y5) contain both non-coding and coding regions, one (Y3) is a subset of a large gene and therefore fully coding, and one (Y4) is from a region with no known genes.

To study correlations between nt sequence and physical size, measurements are best made under solution conditions where the morphology of secondary structures is most evident. We recently showed [8] that the 3D structures of large RNA molecules, when visualized by cryo-electron microscopy in low-ionic-strength/Mg^2+^-free buffers, are consistent with their predicted secondary structures, and that the relative compactness of viral-sequence RNAs observed in these buffers is preserved in higher-ionic-strength Mg^2+^-containing (e.g., physiological) buffers. More explicitly, the presence of Mg^2+^ and higher ionic strength will of course decrease the *absolute sizes* of the RNA molecules, but the radii of gyration for viral-sequence molecules are shown [8] to be significantly smaller than for non-viral sequences in *both* buffers. Therefore, as in our previous studies, we choose in the present work a 10 mM 10∶1 Tris:EDTA (TE) buffer (pH 7.4) as the appropriate solvent for measuring the relative sizes of the RNAs listed above, again accentuating the role of secondary structures under conditions where tertiary interactions are minimal. We are not suggesting that secondary structure is the only important factor in determining the compactness of RNA; tertiary folding effects can of course contribute substantially as well. Rather, we are suggesting that *the extent and nature of branching* in the secondary structure is a dominant factor. Accordingly, our predictions and conclusions relate exclusively to differences in these properties between viral and non-viral sequences.

### Relative Gel Electrophoretic Mobilities

**Figure 1:**
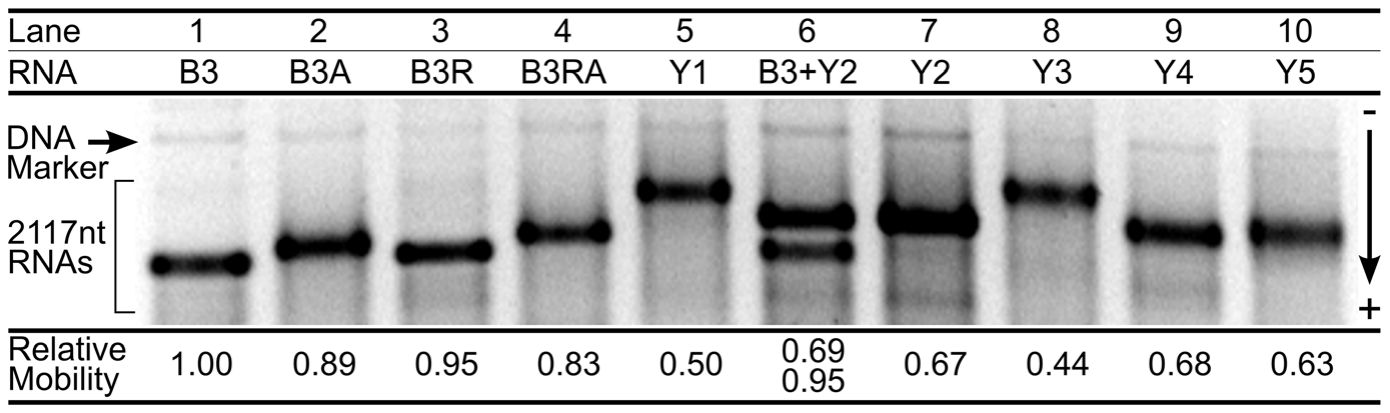
Gel electrophoretic mobilities of 2117-nt RNAs. Lanes 1–4 show a viral RNA (B3) and sequences engineered from it, while lanes 5 & 7–10 show yeast-based transcripts. Each lane contains ≈ 1 *μ*g of RNA, i.e., an ensemble of 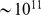 molecules. B3 & Y2 were mixed prior to running in lane 6. Mobility is measured as the distance from the DNA marker (see Methods), and reported relative to B3.

Sizes are first investigated by electrophoresis (Fig. 1) through a 1% agarose gel prepared and run in pH 7.4 TAE buffer (see Methods). Prior to loading, the RNAs were equilibrated in TE buffer for 24 hours to obtain reliable hydrodynamic properties [21]. Each lane contains 1 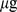 RNA (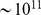 molecules) and 1 ng of a 2141-bp linear dsDNA added as an internal marker.

The RNA band positions in Fig. 1 indicate that B3 (lane 1) has the highest mobility; B3A, B3R and B3RA migrate a slightly shorter distance, whereas the yeast-based sequences Y1–Y5 are most retarded by the gel. The viral and viral-based RNAs are therefore effectively smaller in size compared to Y1–Y5, with B3 being the most compact. To confirm these trends, B3 and Y2 were mixed prior to loading in lane 6. Electrophoresis clearly separates the two bands, demonstrating that the physical properties of their molecular ensembles are distinct. In other words, although each band represents 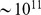 molecules with various secondary and tertiary configurations, the molecular sizes and shapes in a given band (sequence) are closer to each other than to those in other bands. Differences in mobilities have similarly been observed between evolved and random sequences of short (< 100 nt) RNAs [22].

The mobility of an RNA can be quantified as its distance from the DNA marker band (see Methods). Relative mobilities (*μ_r_*) are calculated with respect to the fastest migrating RNA, in this case B3 in lane 1 (see Table S1 in File S1). Because the RNAs all have the same formal charge, differences in their mobilities are expected to arise from differences in their ability to diffuse through the gel network. Relative mobilities therefore represent relative diffusion rates, which in turn are inversely proportional to hydrodynamic radii. To quantify relative hydrodynamic radii in the context of diffusion through an electrophoretic gel, we use the retardation-based effective radius, *R_r_*, defined as the inverse of the mobility, 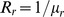.

### Solution Hydrodynamic Radii

**Figure 2:**
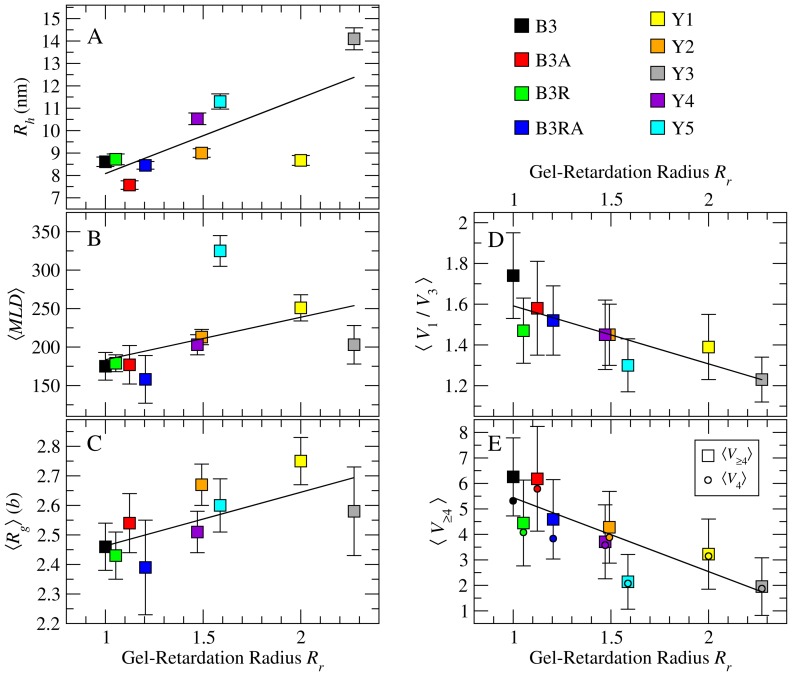
Correlation between measured and predicted size metrics for 2117-nt RNAs. Plotted against gel-retardation radii *R_r_*, are: (A) hydrodynamic radii *R_h_*, (B) ensemble-averaged maximum ladder distance 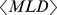, (C) tree-graph radii of gyration *R_g_*, (D) higher-order branching propensity 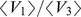, and (E) numbers of *d* = 4 (circles) and *d*≥4 (squares) vertices. Solid lines are least-squares linear regression fits. Error bars are standard deviations (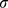) except in A, where they are the standard errors of estimates (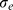). Standard deviations of 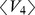 are listed in Table S1 in File S1.

To explore the relationship between gel-electrophoretic retardation and the size of a freely diffusing molecule, solution hydrodynamic radii (*R_h_*) are measured. FCS (fluorescence correlation spectroscopy; see Methods) is used to determine the characteristic time (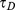) taken by fluorescent RNA molecules to diffuse through a known confocal volume. For a fixed excitation volume, the *R_h_* of a diffusing molecule is directly proportional to 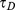. Therefore, comparing the 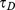 of an RNA with that of a standard allows the quantification of its hydrodynamic radius. Experimental fluorescence auto-correlation curves, and a sample excitation-power series to illustrate the fitting method (see Methods), are shown in Fig. S1 in File S1. *R_h_* values and the standard errors of their estimates are listed in Table S1 in File S1 and plotted against *R_r_* in Fig. 2A.

The linear regression in Fig. 2A reveals a correlation between the values of *R_r_* and *R_h_* for the RNAs. B3 and B3-based RNAs form a group with low retardation and accordingly smaller *R_h_* values. In contrast, the yeast-based sequences generally have higher *R_h_* values. However, the correlation between *R_r_* and *R_h_* is not perfect. While Y3, Y4 and Y5 have increasingly higher *R_h_*s, Y1 and Y2 have unusually low values. Similarly, the trends in *R_r_*s of B3 and B3-derived RNAs (Fig. 1) are not captured by trends in their *R_h_* values.

While recognizing the general correlation between *R_h_* and *R_r_* in Fig. 2A, we reconcile the outliers by acknowledging the inherent shortcomings of measuring the two properties. *R_r_* values implicitly account for deformation and alignment of asymmetric molecules moving in an electrical field through a gel network. These effects are ameliorated by measuring *R_h_*, which represents the radius of an equivalent sphere with the same 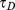 as the molecule. The assumption of spherical geometry, however, is a fundamental limitation of most hydrodynamic measurements, making *R_h_* values less sensitive to small variations in shape and size. For example, gel electrophoresis yields a clear separation in *R_r_* between B3 and Y2, whereas their *R_h_* values are not significantly different. These molecules with similar diffusive properties therefore have sufficiently distinct shapes and sizes to be captured by gel electrophoresis. With the above limitations in mind, we choose *R_r_* as the more sensitive measure of molecular shape and size and try to understand the origin of relative mobilities by analyzing the structural properties of secondary structure ensembles.

### Secondary Structures and Maximum Ladder Distance

Cryo-electron microscopy of large RNA molecules in solution [8] reveals that coarse-grained properties, such as the overall shape and size of an ensemble of molecules in solution, can be deduced using secondary-structure ensembles predicted from the primary nt sequence. This allows us to rationalize the gel-retardation results in terms of the ensemble-averaged properties of predicted secondary structures (see Methods) that best reflect physical shape and size. In an earlier study [7], we considered the longest path along an RNA secondary structure, in terms of the number of base pairs between the ends of the path, as one such physical property. This measure was termed the “maximum ladder distance” (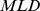) and its average for a Boltzmann ensemble containing 1000 secondary structures determined for a given sequence (see Methods) was represented as 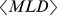. Because 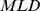 is a measure of the longest physical distance within each secondary structure, we test whether 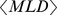 variations between sequences are sufficient to explain their relative gel-retardation rates.


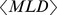
 values computed as described in Methods are listed in Table S1 in File S1 and plotted against *R_r_* in Fig. 2B. Linear regression (solid line) indicates an overall co-variation of 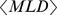 and *R_r_*, but several points (B3RA, Y3 & Y5) are significant outliers. Knowledge of the maximum extents of secondary structures of sequences is therefore not sufficient to reliably predict their relative mobilities. For large RNAs, the 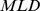 path typically accounts for ≈20% of the molecule's mass. It follows that the details of branching, i.e. how the remaining mass (80%) is distributed about the longest path, play an important role in determining relative mobilities.

### RNA Tree Graphs and Radii of Gyration

The average length of a base-paired helical segment in large RNAs is independent of the length of the sequence [7]. This allows branching patterns in secondary structures to be accurately depicted by tree graphs where helices are represented by edges, and multi-helix junctions and loops by vertices [23]–[25]. (See Fig. S3 in File S1 for example.) This simplification allows statistical measurements developed for ideal branched polymers to be applied to RNA secondary structures. In particular, the radius of gyration (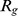) of an equivalent ensemble of ideal polymers with branching patterns identical to an RNA ensemble can be computed using a rigorous theorem due to Kramers [14]. As detailed in Methods, the secondary structure ensemble for each RNA is converted to an ensemble (forest) of tree graphs; the mean radii of gyration (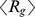
s) are listed in Table S1 in File S1 and plotted against 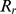 in Fig. 2C. 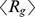 values are in units of edge-length *b*, which represents the mean helix length (≈5 base pairs).

The data and linear regression line in Fig. 2C indicate a general covariation of 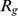 with *R_r_*. As with previous measurements, RNAs that differ significantly in *R_r_* can have similar values of 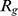 (e.g., Y3 & Y5), and conversely, RNAs with similar *R_r_*s can have significantly different 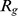
s (e.g., Y2 & Y4). In addition 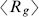 varies at most by 25% over a 2-fold change in *R_r_*, making it a less sensitive measure of differences between sequences. These limitations indicate a need for deeper analysis of the differences in branching patterns.

### Branching Statistics in RNA Trees

Because the degree of a vertex is the number of edges connected to it, the sum of the degrees (*d*) of all the vertices in a graph is twice the total number of edges (*E*). First shown by Euler [26], [27], this relation can be written as
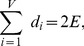
(1)


where 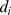 is the degree of the 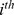 vertex and 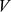 is the total number of vertices in the graph. For tree graphs, where cyclical paths are disallowed by definition, 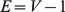. Substituting this equality into Eq. 1 and writing the total number of vertices of each degree *d* as *V_d_* (where 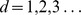), Euler's lemma can be rewritten as 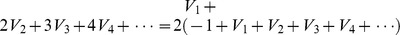. Rearrangement and then division by 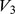 yield the following relations between the numbers of vertices per degree:
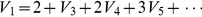
(2)

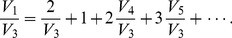
(3)


As shown previously [8], [28], about 95% of vertices in large-RNA tree graphs have degree 1, 2 or 3 (e.g., Fig. S3 in File S1). While 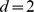 vertices are found in significant numbers, they do not affect the branching, as indicated by the absence of 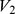 in Eq. 2. The “branchedness” of a tree is ultimately determined by the number of 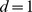 vertices relative to the number of branch points (i.e., 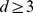 vertices), which further depends on the distribution of vertex degrees. Branching in RNA trees is primarily due to 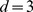 vertices. Higher-order junctions (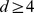), although rare, can make significant contributions to 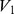 as seen by their progressively higher coefficients in Eq. 2.

For long RNAs, where the 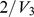 term of Eq. 3 is small, 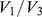 is effectively a constant independent of sequence length (i.e., 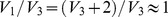) unless higher-order branching is present. As a consequence, we use 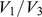 as a convenient length-independent intrinsic measure of higher-order (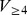) branching propensity. Ensemble-averaged values of this ratio, denoted as 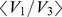, are shown in Table S1 in File S1 for the nine RNAs studied. They are all significantly greater than 1, confirming the presence of 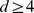 vertices in these RNAs. The values of 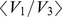 and 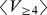 in Table S1 in File S1 (plotted in Figs. 2D & E) confirm that the numbers of vertices of 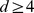 are indeed in the relative order predicted by 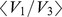 and Eq. 3. Among the 2117-nt RNA ensembles studied, 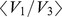 is greatest for B3 (Fig. 2D), suggesting that the viral sequence has the highest propensity for 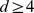 branching.

To understand the trends in 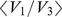, we compare 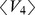 with 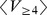 in Fig. 2E (Table S1 in File S1). For all RNAs, we find 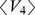 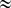 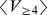, indicating that higher-order vertices are predominantly 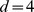. Only B3 (black square) has a significantly higher contribution from 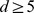 vertices, which stems from the presence of one 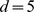 vertex, on average, in every secondary structure in the ensemble. Next, we test if this propensity for higher-order branching is general to all viral genomes by comparing them to random sequences.

### Branching in Random and Other Viral RNAs

**Figure 3:**
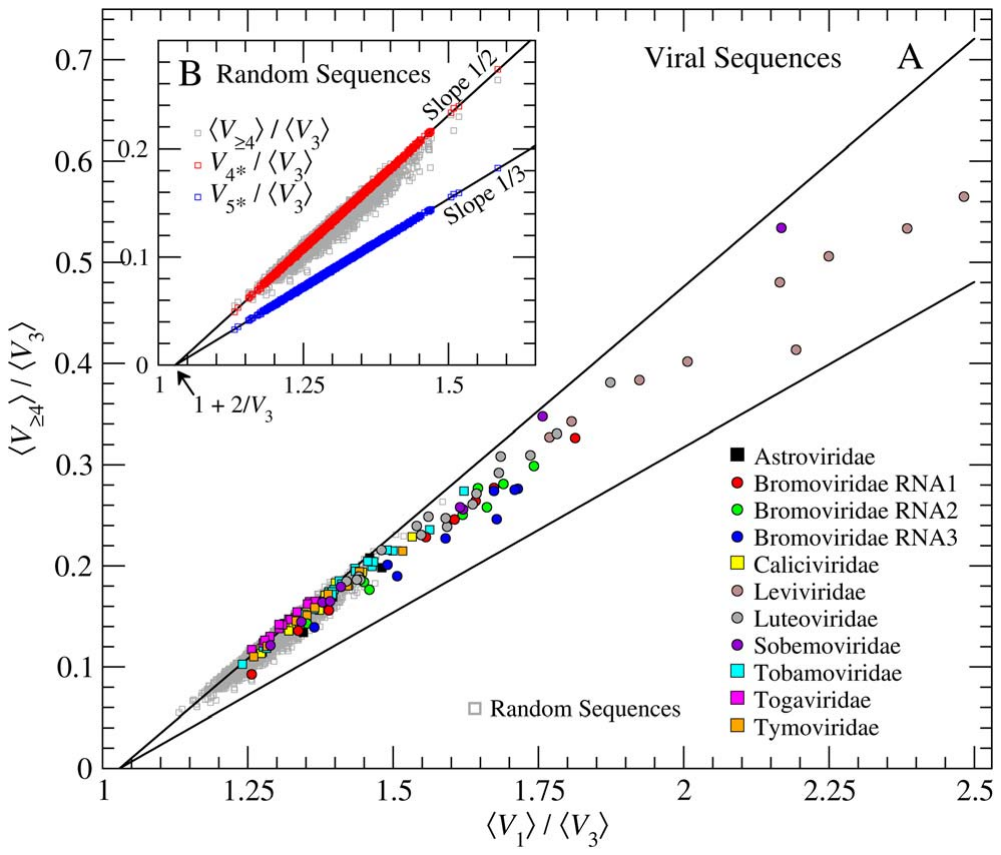
Higher-order branching in random and viral RNAs. 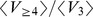
 is shown versus 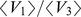 in both plots. Inset B shows 4000-nt random-sequence data (gray squares) with 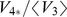 (red squares) and 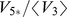 (blue squares) plotted against 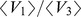 (see Eqs. 4 & 5). Values of 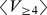/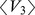 (gray squares) are consistent with 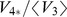, indicating that most higher-order junctions in random RNAs have 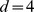. Plot A compares the random sequences with eleven distinct families of viral RNA. Families with more than half their members having 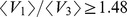 are shown with circular symbols.

To understand the likelihood of higher-order branching in unevolved sequences, we study secondary structure ensembles of 2000 distinct random sequences of length 4000 nt. As with the 2117-nt sequences, the ensemble-averaged numbers of vertices of each degree are determined (see Methods). In Figs. 3A & B, 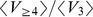, the number of higher-order vertices (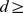
4) per 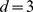 vertex, is plotted against the branching propensity ratio 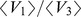. These length-independent ratios facilitate comparison of the random-sequence data with other families of RNAs. Note that values of 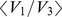 and 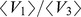 (Table S1 in File S1) are statistically indistinguishable, making the latter an equally good measure of higher-order branching propensity.

Eq. 2 shows that knowing 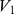 and 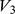, one can estimate the maximum numbers of vertices of higher order. For example, the number of 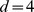 vertices, assuming no higher degree is present, which we call 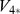, is calculated using
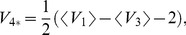
(4)


where the expression in parentheses represents the surplus of 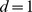 vertices that cannot be explained by the number of 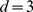 vertices. Similarly, the maximum number of 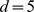 junctions, 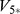, is determined by disallowing vertices of 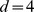 and 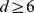:
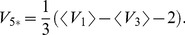
(5)


Ratios of the maximum numbers of junctions to 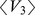 are plotted in Fig. 3B to compare with 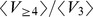. Least-squares linear regression fits to 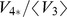 and 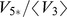, respectively, yield slopes of 1/2 and 1/3 as expected from Eqs. 4 & 5. The 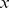-intercept (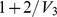) indicates the average number of 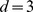 vertices for 4000 nt sequences to be 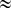 58 (i.e., one 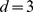 vertex per 69 nt). 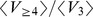 (gray squares), for most sequences, lies along or close to the 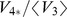 line, indicating that 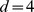 is the dominant form of higher-order branching. Data lying away from this line indicate a small likelihood of randomly generating sequences that lead to junctions with 5 or more helices. However, these do not significantly increase the branching propensity measured by 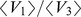. The averages over 2000 random sequences of 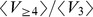 and 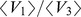 (and their standard deviations) are 0.13 (0.03) and 1.30 (0.06), respectively.

Fig. 3A compares higher-order branching data (Table S2 in File S1) from eleven families of viral RNAs with those from random sequences (Fig. 3B). Astroviridae and Caliciviridae are spherical non-enveloped animal viruses. Bromoviridae are spherical plant viruses containing tripartite genomes (labeled 1, 2 & 3) with each packaging into a separate particle. Leviviridae are spherical non-enveloped viruses that infect bacteria. Luteo-, Sobemo- and Tymoviridae are spherical non-enveloped plant viruses similar to Bromoviridae, but with monopartite genomes. Tobamoviridae constitute a group of rod-like (i.e. filamentous) plant pathogens and Togaviridae are membrane-enveloped animal viruses.

In Fig. 3A, most viral sequences have 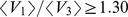, the mean value for random sequences. In fact six of the eleven viral families have members with 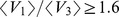, values completely outside the range observed for 2000 random sequences. While the first trend suggests a generally higher propensity for 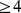-helix loops in viral RNAs, the latter shows that the genomes of some families of viruses have unusually high levels of higher-order branching. Families with half or more of their members with 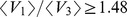 (3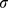 greater than random sequences) are shown with circular symbols and the remaining with squares. It is noteworthy that as 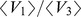 exceeds 1.48, the number of 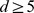 vertices increases, leading to a shift of 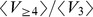 from the 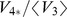 line towards 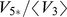.

Thus, knowledge of the number of stem-loops and 3-helix junctions in a secondary structure is sufficient to predict higher-order branching and therefore the compactness of an RNA. These differences in higher-order branching propensities reveal useful details about the patterns of base pairing in the primary sequence. To illustrate this, we analyze the relative proximities of vertices in secondary structure trees and their implications on the information content of the RNA sequence.

### Vertex Distance Distributions and Base Pairing Proximity

To verify that higher-order branching significantly increases the compactness of trees within an ensemble, we compute a graph-distance distribution function 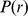 for the nine 2117-nt RNAs (see Fig. S4 in File S1). Analogous to pair-distance distributions from small-angle scattering [29], bell-shaped narrow 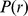
s indicate compact/spherical objects while skewed distributions with long tails represent elongated/anisometric shapes. Instead of a physical distance, 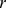 here represents the number of edges (graph-distance) along the tree between pairs of vertices. Fig. S4A in File S1 shows ensemble-averaged 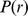
s for the 2117-nt RNAs. Differences in the relative proximity of low-order (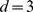) branch points due to higher-order branching can be discerned by computing 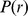 for 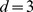 vertices alone (Fig. S4B in File S1). The 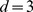 and total 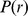 distributions are both significantly narrower for viral (B3) and viral-based RNAs (B3R, B3RA) with comparable gel mobilities and 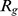
s.

**Figure 4:**
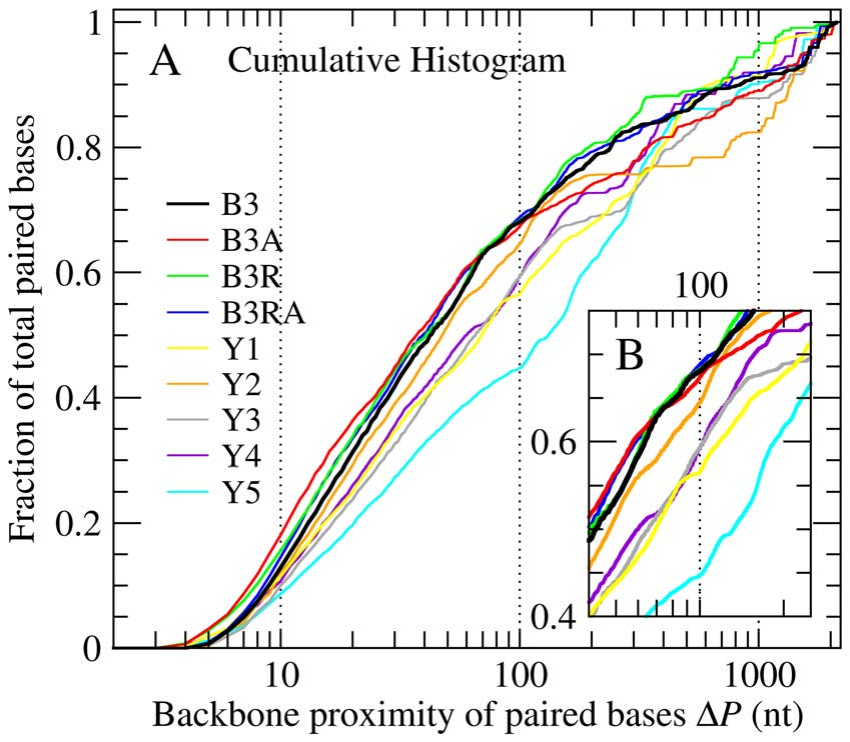
Base-pairing proximity for 2117-nt RNAs. Ensemble-averaged cumulative histograms of backbone distance between paired bases (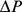) are in F & G. Viral and non-viral histograms diverge up to 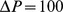 and converge thereafter. Unlike in yeast RNAs, over 70% of base pairs in B3 (See inset G) have 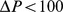. This predominance of proximal base pairing leads to compact secondary structures.

In order for a few 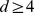 branch points to cause a significant narrowing of the 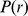 curves, they would need to be placed centrally along the tree so as to increase the density of branched arms while reducing their overall lengths. Shorter arms implicitly contain secondary-structure elements that arise from pairing of bases closer to each other along the RNA backbone. This positional correlation is quantified for each sequence as ensemble-averaged normalized and cumulative histograms (Figs. S5 & 4A) of the backbone distance between paired bases (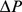). The normalized histograms (Fig. S5 in File S1) for B3 (black curve) and similarly compact RNAs (B3A, B3R & B3RA) are narrower, strongly peaked at 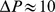, and show less pronounced tails. This is better illustrated by the cumulative histograms (Fig. 4A & B), where nearly 70% of all the base pairs in a secondary structure occur between bases within 100 nt of each other. In comparison, proximal base pairs (i.e. 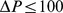 nt) for the yeast sequences can be as few as 45%. As discussed below, this has important implications for the relative stabilities of the kinetically and thermodynamically preferred secondary structures.

## Discussion

This work establishes that differences in the shapes and sizes of long equal-length RNAs in solution can be determined using standard experimental techniques, and explained by the coarse-grained properties of secondary structure ensembles predicted from their sequences. Using graph-theoretical arguments we have shown that knowledge of the number of stem-loops and three-helix junctions in experimentally determined [30] or calculated secondary structure ensembles quantifies the number of higher order multi-helix junctions and therefore the overall compactness and hydrodynamic properties.

Because of the central role played in our analyses by the ensembles of secondary structures associated with different primary sequences, and because the sequences involved are thousands of nts long, it is important to comment on the robustness of our predictions. It is well-known, of course, that all secondary-structure computational algorithms begin to degrade significantly – in their prediction of *base-pairings* – when the sequence lengths begin to exceed several hundred nts. But this failure is not relevant to predicting *coarse-grained* properties of the secondary structures such as vertex order distributions and the extent of higher-order branching, etc., much as we had explicitly shown earlier [7] that relative maximum ladder distances and other size measures of long (thousands-of-nt) sequences do not depend on the details of folding algorithm used. Also, our conclusion – that viral sequences are more compact because their secondary structures are more compact – is not invalidated by our neglect of pseudoknots. Indeed, including the effects of pseudoknots [31] would only make the viral sequences still more compact relative to non-viral ones, because pseudoknots contribute to compaction of an RNA molecule and are more prevalent in viral sequences [32]. Finally, the stability/existence of pseudoknots is favored by Mg^2+^ and high ionic strength [33], and their importance is thus minimized by our choice of TE buffer.

We showed recently [8] that molecular ensembles of large RNAs in solution can generally be represented by a prolate envelope. For RNAs ranging in length from 1000 to 3000 nt, the relative sizes and shapes of molecular envelopes could be distinguished by cryo-EM and explained by the inherent asymmetry of secondary structures and the geometric properties of multi-helix junctions. The present study shows that even RNAs of identical nt length can have significantly different shapes and sizes depending on the density of branching in their secondary structures. Higher-order (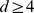) multi-helix junctions represent locations where the density of the molecule is locally high. Larger numbers of higher-order junctions imply the molecule has a higher density and therefore a smaller molecular envelope for the same mass. As seen in Figs. 2D & E, relative densities inferred from numbers of higher-order junctions best explain the gel-mobility trends in Fig. 1.

Bacteriophage MS2 provides a compelling example of the relevance of this kind of analysis to understanding the role of RNA branching statistics in viral assembly. The packaging propensity of MS2 RNA is known [34] to depend on the availability of stem-loops that bind strongly to capsid protein. For a given RNA length, the number of stem-loops in a secondary structure increases with the number of higher-order junctions. This is seen clearly in Table S1 in File S1, where B3 and B3-based sequences consistently show larger numbers of stem-loops (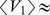 45) and higher-order vertices compared to yeast RNAs. It follows that MS2 RNA, with the highest examined 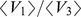 (2.48, see Fig. 3 & Table S2 in File S1), has extremely dense branching that leads to a physically compact molecule with increased numbers of stem-loops for binding protein. Fig. 3 shows that most Leviviridae genomes have 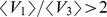, indicating a strong selection for compactness among these bacterial pathogens.

Differences in branching propensities between viral families (Fig. 3, Table ST2) can be understood by analyzing the structural role of the RNA genome in each case. In rod-like viruses such as Tobamoviridae [35], hydrophobic interactions between capsid proteins and electrostatic interactions between RNA and protein supply the energy required to unravel the RNA secondary structure and confine the genomic molecule within a thin long cylindrical volume. Due to the restructuring of RNA, the compactness of the genomic molecule before its packaging is not relevant to the survival of these viruses. Accordingly we find that values of 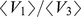 in the Tobamoviridae family (Fig. 3, cyan squares) are not significantly different from random sequences. Compactness becomes important when the genome needs to be packaged in a limited spherical volume.

The evolutionary pressure for compactness is best understood by comparing the genomes of spherical viruses of similar sizes and triangulation (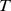) numbers [36]. Seven of the nine families in Fig. 3 (Astroviridae, Bromoviridae, Caliciviridae, Leviviridae, Luteoviridae, Sobemoviridae and Tymoviridae) contain viruses with spherical capsids of similar diameters (27-30 nm) and internal volumes. The capsid in each case exhibits 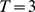 icosahedral symmetry, and is composed of 180 copies of identical coat proteins. One way to condense RNA molecules to a size comparable to their capsids is to use condensing agents. Just as linear anionic DNA molecules condense into densely packed toroids or aggregates in the presence of polycations such as spermine and spermidine [37], [38], individual RNAs molecules are known to acquire a physically compact state in the presence of natural polyamines [39], [40]. Hundreds of molecules of spermidine [41] are known to condense the genomic RNA molecule in Tymoviridae. Similarly, Caliciviridae RNAs are condensed by small basic proteins produced by the translation of their viral genomes [42], [43]. If condensation is caused mainly by polyamines or basic polypeptides, there should be minimal pressure on the viral genomes to be densely branched and intrinsically compact. Consistent with this, Caliciviridae (Fig. 3, yellow squares) and Tymoviridae (Fig. 3, orange squares) genomes do not show significantly higher propensities for branching than random sequences.

Condensing agents are not found in the other five families of 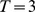 viruses studied. In the absence of polycations, the condensation and confinement of RNA genomes can also be achieved by their electrostatic interaction with basic residues often present on disordered protein tails on the inner surface of the capsid [44]. Because the capsids of these viruses contain the same number of coat proteins and therefore the same number of positively charged internal tails, the degree of electrostatic stabilization of the genome depends on the length, flexibility and the number of basic residues on each tail. In other words, viruses with fewer positive charges on their internal protein tails should rely more on the intrinsic compactness of the RNA genome for stability. The number of basic residues on the RNA-accessible N-terminal disordered tails in Astroviridae are typically between 20 and 30 [45]. For Bromoviridae [44], [46]–[48], Luteoviridae [49], [50] and Sobemoviridae [51], this number ranges between 10 and 20, whereas Leviviridae coat proteins do not have charged tails [34], [52]. Consistent with the above prediction, we find (see Fig. 3) that Astroviridae genomes (black squares) deviate least from random sequences in their branching propensity, Bromoviridae (red, green & blue circles), Luteoviridae (gray circles) and Sobemoviridae (magenta circles) genomes typically have values of 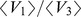 more than one standard deviation higher than random sequences, and Leviviridae RNAs (brown circles) are the most densely branched.

The family Togaviridae consists of membrane-enveloped 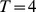 viruses. Besides being physically larger, their capsids consist of 240 copies of identical coat proteins, each with 10 to 15 RNA-accessible basic residues. Comparing Togaviridae to 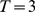 Bromoviridae, whose coat proteins have similar numbers of RNA-accessible basic residues, allows us to evaluate the impact of a larger size on the selective pressure for RNA compactness. For example, Sindbis virus has a genome nearly 4 times as long as the RNA inside CCMV virions and their capsid proteins have around 10 basic residues each [46], [53] in the N-terminal disordered regions. Theoretical models [7], [14] and a comparison of the sizes of RNAs in the PDB database [15] indicate that the 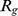
s of RNA molecules scale as 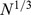, where 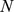 is the number of nucleotides in the sequence. Their volumes should therefore be directly proportional to the nt length of the sequence. The Sindbis genome therefore occupies nearly four times the volume of say CCMV RNA1. Whether this represents a greater need for compaction can be discerned by comparing their internal volumes. The internal radii of capsids of Sindbis and CCMV are 18.2 [54] and 9.4 nm [55], respectively, which means the internal volume of Sindbis is nearly eight times that of CCMV. Because the RNA volume increases by a smaller factor than the internal volume, we expect the Sindbis genome to be under less pressure to be compact than a CCMV RNA. The compactness requirement is further reduced because a 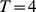 capsid contains 4/3 times more coat proteins and RNA-exposed basic residues than a 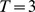 one. It is therefore not surprising that the branching propensities of Togaviridae genomes (Fig. 3, pink squares) are lower than those of Bromoviridae such as CCMV, and indistinguishable from those of random sequences.

As seen above, estimating the pressure for RNA compactness by evaluating electrostatic stabilization provides insight into the relative branching propensities of various virus families. While genome sequences are available for most known viruses [56], high-resolution capsid structures and the nature of interaction between the RNA and capsid proteins are known for far fewer [57]. As more structural details emerge, sophisticated models that include additional factors – such as variations in internal volumes or the presence of basic residues in VPg [58], [59], a protein covalently bound to the 5′ end of the RNA in many viruses – can be used to clarify further the selective pressure for viral RNA compactness.

The information leading to the compactness of branching is ultimately encoded in the sequence of nucleotides in the primary sequence. We illustrate this in Fig. 4A by introducing the quantity 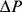 as a metric that reflects positional correlations of pairable (complementary) base patterns along the primary sequence. It is particularly notable that although the three B3-derived RNAs formally differ in sequence from B3, each retains the local availability of pairable nt patterns. Large-scale changes like gene-swapping or changing the sense of the strand conserve the relative positions of locally available pairable regions. The fact that compactness is preserved in these sequences (Fig. 1) indicates that it is encoded on a scale smaller than the length of either gene in B3 (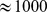 nt). The consistently larger sizes of Y1–Y5, irrespective of whether they are non-, partially- or fully-coding, indicate that the signature for compactness does not depend on whether the RNA codes for a protein. Rather, it involves strong proximal base pairing (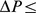 100 nt), as seen in Fig. 4B for B3-based but not for yeast-based RNAs. The distinguishing length scale of 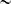
100 nt, while much larger than that of a canonical stem-loop (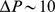), is only slightly larger than that of a three-helix junction (recall that random sequences produce on average a 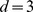 vertex every 69 nt). Increased non-trivial local base pairing, also observed in some translated bacterial RNAs [60], has important effects on RNA folding.

The strong proximal pairing identified above for viral and related sequences is based on ensembles of free-energy-minimized secondary structures. This represents a very unusual case, where the global minimum free-energy structure heavily relies on local base pairing – up to 75% within 100 nt, as seen in Fig. 4B. In other words, if we were to predict secondary structures for the same sequences with the limitation of local pairing [61]–[63], we would recover most of the branching seen in the globally minimized structure without a significant free-energy cost. Locally-folded secondary structures represent a scenario where folding is kinetically quenched, i.e., co-transcriptional [64]–[66]. Denser branching and stronger proximal pairing thus ensure similar folding outcomes for viral sequences under thermodynamic and kinetically controlled conditions. Robustness of the structural outcome of viral-RNA folding to variations in the environment represents an evolutionary advantage – that of the reliable packaging of the genome into nanoscopic protein capsids. This advantage often works in parallel with specific local secondary and tertiary structure motifs associated with short sequences essential for genome packaging in many RNA viruses [67], [68].

While we have concerned ourselves exclusively in the present work with ssRNA viruses, similar arguments should apply as well to ssDNA viruses for which the genome is co-self-assembled with capsid protein in spherical shells. We have focused on ssRNA because these viruses are so much more prevalent, involving a wide variety of well-known pathogens whose host ranges include bacteria, plants, and animals. One reason for spherical viruses needing to be small is simply so that larger numbers of them can fit into their host cell before it lyses or is otherwise obliged to shut down viral synthesis. In the case of plant viruses, which spread to neighboring cells through the plasmodesmata channels traversing cell walls, the capsid diameter is still more severely constrained; indeed, in many instances, a viral gene is dedicated to a protein product that chaperones virus particles to surrounding cells by reorganizing the otherwise-too-small plasmodesmata. For this reason, even rod-like viruses, whose RNA genomes are not under pressure to be compact, must still have the smallest dimension (cross-sectional diameter) of their capsids sufficiently small.

By demonstrating i) that experimentally determined RNA sizes are related to the compactness of branching patterns in secondary structure ensembles, and ii) that the compactness of several families of viral genomes are consistent with the selective pressures imposed by capsid size and electrostatics, we have shown that the density of secondary-structure branching is a degree of freedom available for optimization in viral RNA genomes. When other means of condensing the genome are absent, viral RNAs are unusually compact.

## Methods

### RNA Synthesis and Purification

The RNA molecules were *in vitro* transcribed from PCR templates using T7-polymerase (courtesy of Prof. Feng Guo, UCLA), followed by DNAse digestion of the template. Protein impurities were removed by phenol-chloroform extraction and the RNA isolate was rid of shorter polynucleotides and unreacted ribonucleotides by repetitive additions of TE (pH 7.4) buffer and filtration through a 100 kDa MWCO Centricon device. RNA samples were equilibrated in TE buffer at 4°C for 24 hours to obtain uniform ensembles [8], [21] and typically used within 48 hours of preparation. DNA templates for B3, B3A, B3R and B3RA were amplified by PCR from linearized plasmids of B3 and B3R [20] by designing appropriate primers. The templates for Y1-5 were similarly made by PCR from genomic yeast DNA. The sequence from the second base onwards for Y1, Y2, Y3, Y4 & Y5 correspond to those starting from the 855700th, 874269th, 353947th, 390695th and 687701st base of chromosome XII of *Saccharomyces cerevisiae* [69]; note that the first base of a T7-polymerase transcript is required to be a G. These five yeast sequences have nucleotide compositions, and hence fractions of bases paired, comparable to those of the four viral-derived RNAs. A formaldehyde denaturing gel [70] confirmed that the nine RNA transcripts had identical nucleotide lengths (see Fig. S2 in File S1). Fluorescent RNAs – used in our fluorescence correlation spectroscopy (FCS) experiments – were synthesized by spiking the transcription reaction mixture with ChromaTide Alexa Fluor 488-5-UTP (Life Technologies, Carlsbad, CA) such that 5 in every 1000 NTP (nucleotide triphosphate) molecules were fluorescently tagged. Due to lower inclusion efficiency of the fluorescent UTP compared to the untagged nucleotide, the 2117-nt RNA transcripts contained either none or just one Alexa-488 tag at a randomly chosen UTP position. This is desirable because untagged molecules are not counted in FCS, however multiple tagging can lead to higher apparent concentrations. Tagging efficiency of 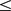
1 was verified by comparing FCS profiles of known concentrations of RNAs and standards.

### Gel Electrophoresis & Mobility Measurements

About 1 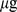 of equilibrated RNA in 10 mM TE buffer (pH 7.4), mixed with 1 ng of 2142 base-pair dsDNA marker, was loaded in each lane. The 1% native agarose gel was prepared and run at room temperature in TAE buffer (pH 7.4). It was stained with ethidium bromide for 20 minutes and the excess stain rinsed away prior to imaging to minimize background fluorescence. The gel was recorded as a TIFF image and imported into ImageJ [71] for mobility analyses. The gel analysis plugin was used to generate one-dimensional mobility profiles from the fluorescence image. Individual mobilities were measured as the distance in pixels between the peak maxima of the RNA and marker bands. The mobility of an RNA divided by that of B3 is used as the relative mobility (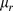).

### Fluorescence Correlation Spectroscopy (FCS)

The Advanced Light Microscopy shared facility at the California NanoSystems Institute (UCLA) was used for fluorescence correlation. The setup contains a custom-made confocal configuration built with an Axiovert 100 (Zeiss, Germany) inverted microscope as its base. The 488-nm line from a continuous-wave Argon Laser (Ion Laser Technology, Frankfort, IL) was used with excitation power ranging from 5–90 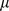
W. A water immersion objective (1.2 NA, 63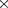, Zeiss) was used in combination with a 50-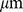 pinhole to achieve an excitation volume of 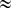 1 fl. Between 7–10 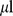 of RNA sample were sealed between two 150-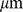 glass slides using silicone isolators (Grace Bio-labs, Bend, OR) and placed on the microscope stage for imaging. Fluorescence signal was collected with the focal volume 35 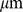 away from the glass surface to prevent substrate interactions. The signal was split evenly to two APDs (AQR-14, Perkin-Elmer Inc) and the channels cross correlated with a temporal resolution of 6.5 ns using an ALV-6010 correlator (ALV GmbH, Langen, Germany). Auto-correlation curves, 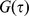, were first obtained for Alexa Fluor 488 (Life Technologies, Carlsbad, CA) in TE buffer, which was used as a size standard of known diffusion constant [72], [73]. Four curves with progressively higher excitation powers between 10 and 90 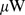 were globally fit to obtain the characteristic diffusion time (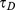) and triplet relaxation time (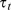) using the following 2D diffusion model for a Gaussian excitation volume [74], [75]:
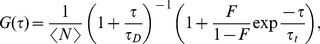
(6)


where 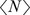, the time averaged number of fluorescent molecules in the focal volume, and 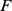, the fraction of molecules in the triplet state, are constant for a sample at a fixed excitation power. The variables 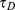 and 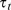 were fit globally (Origin 7, OriginLab, Northampton, MA) to the excitation-power series while allowing 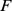 to have distinct values for each power. Fitted curves for a sample RNA molecule (B3) are shown in Fig. S1A in File S1. Values of 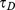 were similarly obtained for the remaining RNA molecules. The diffusion constant of Alexa Fluor 488 is measured to be 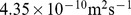 [73]; its hydrodynamic radius (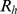) was calculated using the Einstein-Stokes relation to be 0.50 nm in aqueous media. Knowing 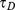 values for both the standard dye and RNA samples, values of 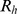 in Table S1 in File S1 are computed using the relation 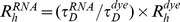. Concentration-normalized auto-correlation curves at 15 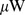 excitation power for the nine RNAs and Alexa Fluor 488 standard are shown in Fig. S1B in File S1.

### Secondary Structure Prediction and Tree Graph Analyses

RNA primary sequences were obtained from the NCBI Viral Genome project [56]. Boltzmann-sampled secondary structure ensembles with 1000 configurations of each RNA were calculated using the RNAsubopt program in Vienna [76] as described in earlier work [7]. Each secondary structure in the ensemble was then converted using the RNA-As-Graphs program [23], [24] into the Laplacian matrix representing the corresponding tree graph. Adjacency and degree matrices were deduced from the Laplacian for further analyses. Degree matrices were used to calculate 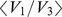, 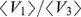, 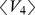, 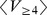, etc (see Results). The adjacency matrices were analyzed using the Mathematica Graph Utilities package and custom programs to compute the graph-pair distribution functions [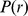] shown in Fig. S4 in File S1. The radius of gyration, 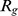, for each tree graph was computed using Kramers' method as described in Ref. [14].

## Supporting Information

File S1 **Combined Supporting Information.** Single PDF file containing Figures S1-S5 and Tables S1 & S2. Legends are provided within the file below each Figure or Table.
(PDF)
