# Supplementary material for: Viral RNAs Are Unusually Compact
Source: PLoS One. 2014 Sep 4;9(9):e105875. doi: 10.1371/journal.pone.0105875 (PMC4154850; doi:10.1371/journal.pone.0105875)
Supplement: File S1 — Combined Supporting Information. Single PDF file containing Figures S1-S5 and Tables S1 & S2. Legends are provided within the file below each Figure or Table. (PDF) [file pone.0105875.s001.pdf]

ID	Measured		Calculated from Secondary Structure Ensembles									
	μ_r	R_h, nm	$\langle MLD \rangle$	$\langle R_g \rangle$, b	$\langle V_1 \rangle$	$\langle V_2 \rangle$	$\langle V_3 \rangle$	$\langle V_4 \rangle$	$\langle V_5 \rangle$	$\langle V_{\geq 4} \rangle$	$\frac{\langle V_1 \rangle}{\langle V_3 \rangle}$	$\langle \frac{V_1}{V_3} \rangle$
B3	1.00	8.61	175	2.46	41.42	64.59	25.19	5.32	0.73	6.26	1.64	1.74
		(0.21)	(18)	(0.08)	(2.65)	(5.34)	(2.84)	(1.57)	(0.76)	(1.53)	(0.21)	(0.21)
B3A	0.89	7.57	177	2.54	47.09	56.31	31.09	5.79	0.34	6.18	1.51	1.58
		(0.19)	(25)	(0.10)	(2.53)	(5.54)	(3.44)	(2.04)	(0.55)	(2.06)	(0.19)	(0.23)
B3R	0.95	8.72	179	2.43	43.01	56.97	30.38	4.08	0.26	4.45	1.42	1.47
		(0.24)	(11)	(0.08)	(1.91)	(4.04)	(2.72)	(1.50)	(0.50)	(1.68)	(0.14)	(0.16)
B3RA	0.83	8.45	158	2.39	45.13	62.27	31.07	3.83	0.73	4.59	1.45	1.52
		(0.17)	(31)	(0.16)	(2.25)	(4.86)	(2.96)	(1.52)	(0.74)	(1.56)	(0.16)	(0.17)
Y1	0.50	8.67	251	2.75	34.65	73.59	25.10	3.14	0.08	3.23	1.38	1.39
		(0.22)	(17)	(0.08)	(1.97)	(4.80)	(2.65)	(1.35)	(0.27)	(1.38)	(0.17)	(0.16)
Y2	0.67	9.00	213	2.67	43.97	57.59	31.19	3.88	0.39	4.28	1.41	1.45
		(0.19)	(10)	(0.07)	(1.72)	(4.05)	(2.71)	(1.36)	(0.57)	(1.41)	(0.13)	(0.15)
Y3	0.44	14.10	203	2.58	37.59	63.56	30.53	1.87	0.08	1.95	1.23	1.23
		(0.49)	(25)	(0.15)	(2.01)	(5.62)	(2.57)	(1.12)	(0.28)	(1.13)	(0.12)	(0.11)
Y4	0.68	10.53	203	2.51	36.62	60.92	27.21	3.58	0.13	3.71	1.35	1.45
		(0.26)	(13)	(0.07)	(1.80)	(4.22)	(2.81)	(1.43)	(0.35)	(1.45)	(0.15)	(0.17)
Y5	0.63	11.30	325	2.60	29.48	73.88	22.54	2.07	0.06	2.14	1.31	1.30
		(0.34)	(20)	(0.09)	(2.10)	(5.07)	(2.12)	(1.07)	(0.25)	(1.07)	(0.15)	(0.13)

Table S1: Structural properties of 2117-nt viral and non-viral RNAs. Numbers below the R_h values (in parentheses) are the standard errors of the estimate (σ_e) for the global FCS fit (see Methods, Fig. S1A). Numbers in parentheses below all other values are standard deviations (σ).

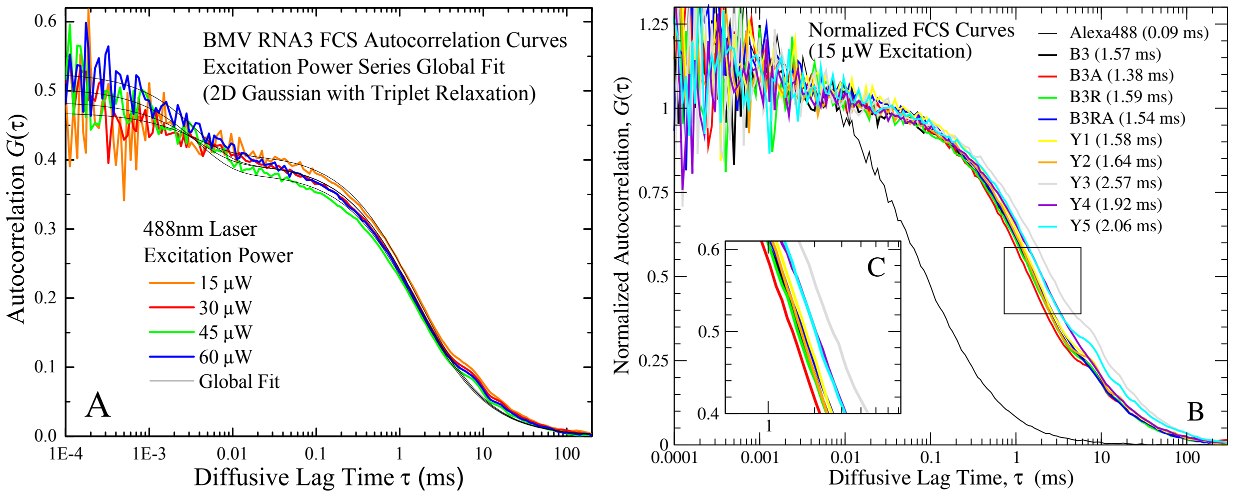

Figure S1: Sample excitation power series for B3 (A), and normalized FCS curves for 2117-nt RNAs (B & C) with τ_D values in parentheses. Solid lines in A are global fits (see Methods).

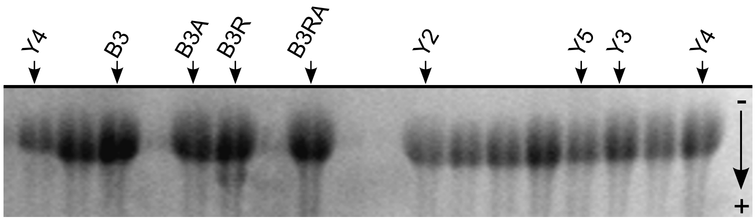

Figure S2: Formaldehyde Denaturing Gel-Eletrophoresis of 2117-nt RNAs. The mobilities of several RNAs are identical under denaturing conditions confirming their identical lengths. The unmarked lanes are additional 2117-nt yeast-based transcripts not presented in this study. Ethidium bromide was used for staining the gel.

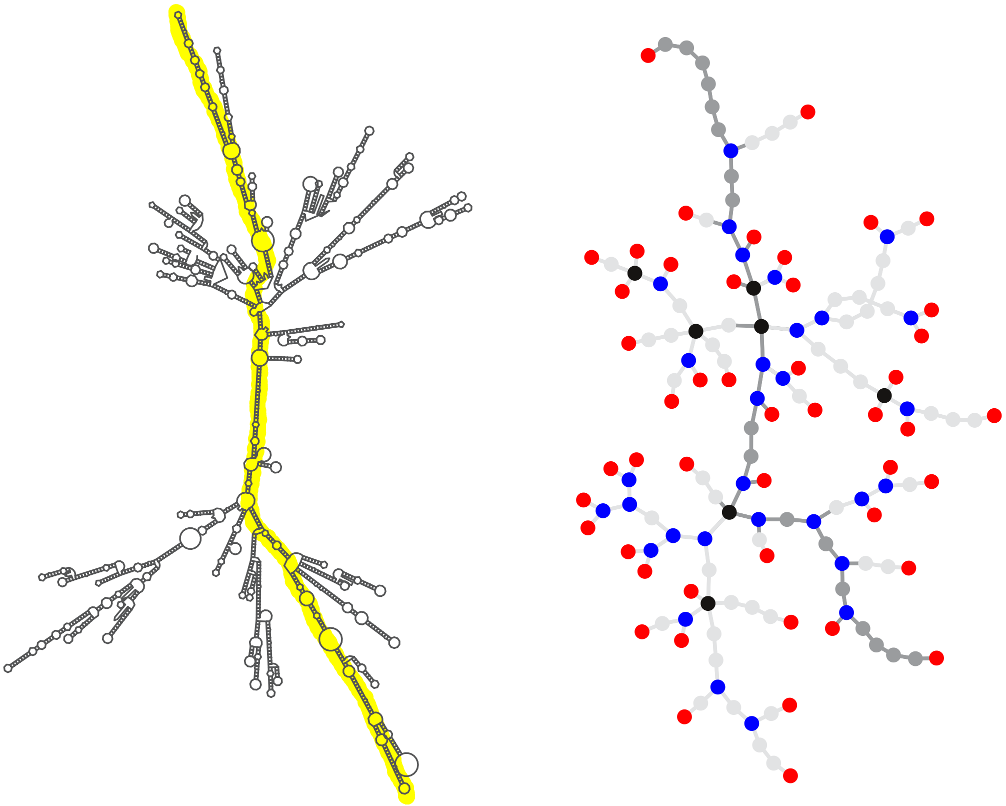

Figure S3: Comparison of a predicted secondary structure of a 2774-nt RNA (CCMV RNA2) and its corresponding tree graph. ● $V_2 = 64$,● $V_1 = 48$,● $V_3 = 30$,● $V_{\geq 4} = 7$. Typically $\geq 95\%$ vertices are $d \leq 3$. The *MLD* path is highlighted in yellow in the secondary structure and with dark gray vertices in the equivalent tree.

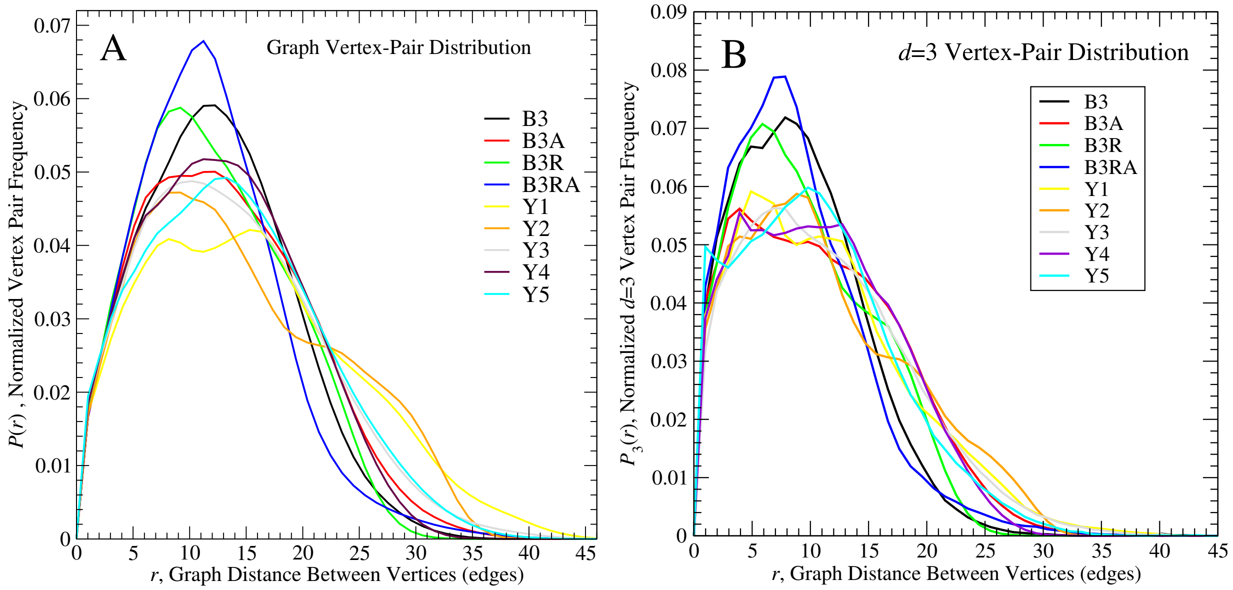

Figure S4: Graph Distance Distributions, $P(r)$, for all vertices (A) and only $d = 3$ vertices (B).

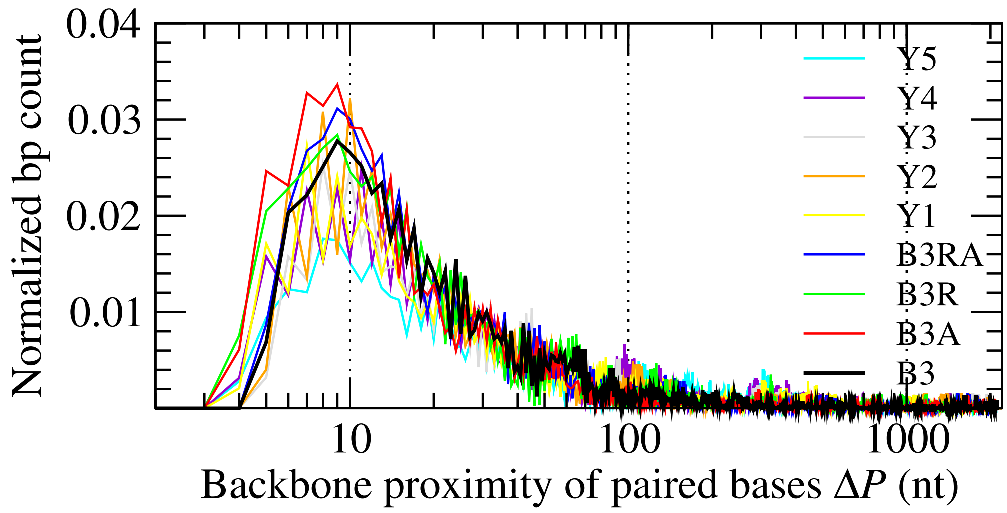

Figure S5: Ensemble-averaged normalized histograms of pairing proximity in 2117-nt ensembles. The fraction of total number of bases in an ensemble of 1000 secondary structures with a given ΔP is shown against the logarithm of ΔP. The peak extending to ΔP ~100 represents recurrent motifs formed by bases within 100 nt from each other. Yeast based RNAs generally have longer tails as seen in the cumulative histogram (Fig. 4A).

NCBI ID	Virus/Genome Name	nt	$\langle V_1 \rangle$	$\langle V_3 \rangle$	$\langle V_{\geq 4} \rangle$	$\langle V_1 \rangle / \langle V_3 \rangle$	$\langle V_{\geq 4} \rangle / \langle V_3 \rangle$
**Astroviridae**							
NC_005790	Turkey astrovirus 2	7355	132.8	98.7	13.2	1.35	0.13
NC_003790	Chicken astrovirus	6927	135.9	91.8	18.2	1.48	0.20
NC_001943	Human astrovirus	6813	127.5	96.3	13.7	1.32	0.14
NC_004579	Mink astrovirus	6610	131.4	90.0	18.7	1.46	0.21
NC_002469	Ovine astrovirus	6440	111.9	80.2	13.6	1.40	0.17
NC_011400	Astrovirus MLB1	6171	108.0	84.7	9.7	1.28	0.11
**Bromoviridae RNA1**							
NC_004008	Broad bean mottle virus RNA 1	3158	58.7	42.2	6.6	1.39	0.16
NC_003543	Cowpea chlorotic mottle virus RNA 1	3171	58.5	37.6	8.6	1.56	0.23
NC_006999	Cassia yellow blotch virus RNA1	3178	64.4	35.5	11.6	1.81	0.33
NC_002026	Brome mosaic virus RNA 1	3234	64.5	40.2	9.9	1.61	0.25
NC_004120	Spring beauty latent virus RNA 1	3252	61.7	36.9	10.2	1.67	0.28
NC_002034	Cucumber mosaic virus RNA 1	3357	65.8	49.2	6.7	1.34	0.14
NC_002038	Peanut stunt virus RNA 1	3357	69.0	54.9	5.1	1.26	0.09
NC_003837	Tomato aspermy virus RNA 1	3410	70.2	42.8	11.3	1.64	0.26
**Bromoviridae RNA2**							
NC_007000	Cassia yellow blotch virus RNA2	2720	53.6	31.7	8.9	1.69	0.28
NC_003541	Cowpea chlorotic mottle virus RNA 2	2774	49.0	33.6	5.9	1.46	0.18
NC_004007	Broad bean mottle virus RNA 2	2799	55.9	32.1	9.6	1.74	0.30
NC_002027	Brome mosaic virus RNA 2	2865	56.8	35.1	8.8	1.62	0.25
NC_004121	Spring beauty latent virus RNA 2	2898	56.6	34.0	8.8	1.66	0.26
NC_002039	Peanut stunt virus RNA 2	2947	62.5	43.1	7.9	1.45	0.18
NC_002035	Cucumber mosaic virus RNA 2	3050	61.6	37.4	10.4	1.65	0.28
NC_003838	Tomato aspermy virus RNA 2	3074	60.7	44.9	6.4	1.35	0.14
**Bromoviridae RNA3**							
NC_007001	Cassia yellow blotch virus RNA3	2091	42.3	24.7	6.8	1.71	0.28
NC_002028	Brome mosaic virus RNA 3	2117	41.0	24.5	6.7	1.67	0.27
NC_003542	Cowpea chlorotic mottle virus RNA 3	2173	41.9	24.5	6.7	1.71	0.28
NC_004122	Spring beauty latent virus RNA 3	2213	43.9	27.6	6.3	1.59	0.23
NC_004006	Broad bean mottle virus RNA 3	2293	43.0	31.5	4.4	1.36	0.14
NC_002040	Peanut stunt virus RNA 3	2188	44.1	29.6	5.9	1.49	0.20
NC_001440	Cucumber mosaic virus RNA 3	2216	46.2	30.7	5.8	1.51	0.19
NC_003836	Tomato aspermy virus RNA 3	2386	53.9	32.1	7.9	1.68	0.25
**Caliciviridae**							
NC_011704	Rabbit calicivirus Australia 1 MIC-07	7422	129.9	97.5	13.6	1.33	0.14
NC_002615	European brown hare syndrome virus	7442	128.9	101.2	12.0	1.27	0.12
NC_011050	Steller sea lion vesivirus	8305	155.6	113.3	17.7	1.37	0.16
NC_008311	Murine norovirus 1	7382	137.1	102.9	15.3	1.33	0.15
NC_006554	Sapovirus C12 strain C12	7476	144.4	106.9	16.5	1.35	0.15
NC_006269	Sapovirus Hu/Dresden/pJG-Sap01/DE	7429	142.1	107.3	15.0	1.32	0.14
NC_010624	Sapovirus Mc10	7458	141.2	107.6	15.4	1.31	0.14
NC_002551	Vesicular exanthema of swine virus	8284	154.9	107.2	20.6	1.44	0.19
NC_001959	Norwalk virus	7654	140.1	110.1	12.5	1.27	0.11
NC_001543	Rabbit hemorrhagic disease virus-FRG	7437	132.6	95.9	16.4	1.38	0.17
NC_001481	Feline calicivirus	7683	129.8	89.7	17.4	1.45	0.19
NC_000940	Porcine enteric calicivirus	7320	143.2	108.5	14.7	1.32	0.14
NC_004542	Canine calicivirus	8513	149.9	97.8	22.4	1.53	0.23
NC_004541	Walrus calicivirus	8289	145.7	102.4	18.5	1.42	0.18
NC_007916	Newbury agent 1 virus	7454	144.4	105.1	17.3	1.37	0.16
NC_006875	Calicivirus isolate TCG	7453	135.0	105.7	12.6	1.28	0.12
NC_008580	Rabbit vesivirus	8380	152.1	109.3	18.6	1.39	0.17
NC_004064	Calicivirus strain NB	7453	141.0	100.7	18.4	1.40	0.18
**Leviviridae**							
NC_008294	Pseudomonas phage PRR1	3573	59.9	27.3	11.3	2.19	0.41
NC_001333	Enterobacteria phage fr	3575	71.9	35.8	14.4	2.01	0.40
NC_002700	Acinetobacter phage AP205	4268	82.0	42.6	16.3	1.92	0.38
NC_002250	Enterobacteria phage KU1	3486	64.9	27.2	14.5	2.38	0.53
NC_001628	Pseudomonas phage PP7	3588	60.2	26.7	13.5	2.25	0.51
NC_001426	Enterobacteria phage GA	3466	60.6	33.6	11.5	1.81	0.34
NC_001417	Enterobacterio phage MS2	3569	73.0	29.4	16.6	2.48	0.56
NC_004301	Enterobacteria phage FI	4276	90.0	50.9	16.6	1.77	0.33
NC_001890	Enterobacteria phage Qbeta	4215	78.4	36.2	17.4	2.17	0.48
**Luteoviridae**							
NC_004751	Cereal yellow dwarf virus-RPV	5723	118.2	74.2	17.7	1.59	0.24
NC_004750	Barley yellow dwarf virus - PAV	5677	117.8	81.8	15.5	1.44	0.19
NC_003491	Beet mild yellowing virus	5722	129.7	77.1	22.5	1.68	0.29
NC_008249	Chickpea chlorotic stunt virus	5900	121.4	73.9	20.0	1.64	0.27
NC_006265	Carrot red leaf virus	5723	118.3	79.9	17.2	1.48	0.22
NC_004756	Beet western yellows virus	5666	125.3	78.8	19.5	1.59	0.25
NC_004666	Barley yellow dwarf virus - GAV	5685	119.7	77.7	18.6	1.54	0.24
NC_003743	Turnip yellows virus	5641	129.8	77.0	23.7	1.69	0.31
NC_003688	Cucurbit aphid-borne yellows virus	5669	125.2	76.5	19.9	1.64	0.26
NC_003680	Barley yellow dwarf virus - MAV	5273	108.5	75.2	14.0	1.44	0.19
NC_003629	Pea enation mosaic virus-1	5706	110.6	76.9	14.3	1.44	0.19

continued on next page

NCBI ID	Virus/Genome Name	nt	$\langle V_1 \rangle$	$\langle V_3 \rangle$	$\langle V_{\geq 4} \rangle$	$\langle V_1 \rangle / \langle V_3 \rangle$	$\langle V_{\geq 4} \rangle / \langle V_3 \rangle$
NC_003369	Bean leafroll virus	5964	117.7	76.0	17.5	1.55	0.23
NC_003056	Soybean dwarf virus	5853	115.8	74.2	18.4	1.56	0.25
NC_002766	Beet chlorosis virus	5776	127.5	73.5	22.7	1.74	0.31
NC_002198	Cereal yellow dwarf virus-RPS	5662	121.3	68.1	22.5	1.78	0.33
NC_002160	Barley yellow dwarf virus-PAS	5695	115.5	81.3	15.0	1.42	0.18
NC_001747	Potato leafroll virus	5987	127.9	68.2	26.0	1.87	0.38
● Sobemoviridae							
NC_002568	Sesbania mosaic virus	4148	71.2	50.5	9.0	1.41	0.18
NC_003747	Ryegrass mottle virus	4210	78.8	48.6	12.4	1.62	0.26
NC_004346	Subterranean clover mottle virus	4258	82.2	59.6	9.8	1.38	0.16
NC_004553	Turnip rosette virus	4037	75.9	58.9	7.1	1.29	0.12
NC_004060	Southern bean mosaic virus	4136	73.2	41.6	14.5	1.76	0.35
NC_002618	Cocksfoot mottle virus	4082	70.8	43.8	11.3	1.62	0.26
NC_001696	Lucerne transient streak virus	4275	79.1	58.9	8.5	1.34	0.14
NC_001625	Southern cowpea mosaic virus	4194	72.5	52.1	8.6	1.39	0.16
NC_001575	Rice yellow mottle virus	4450	82.4	38.0	20.3	2.17	0.53
■ Tobamoviridae							
NC_001801	Cucumber green mottle mosaic virus	6424	104.2	71.2	14.2	1.46	0.20
NC_009642	Bell pepper mottle tobamovirus	6375	106.2	74.0	14.6	1.43	0.20
NC_002692	Tomato mosaic virus	6383	100.0	64.0	15.1	1.56	0.24
NC_001556	Tobacco mild green mosaic virus	6355	91.0	65.7	10.8	1.39	0.17
NC_009041	Rehmannia mosaic virus	6395	107.7	79.0	12.4	1.36	0.16
NC_008310	Hibiscus latent Singapore virus	6474	106.0	70.7	15.2	1.50	0.21
NC_003630	Pepper mild mottle virus	6357	103.0	73.6	13.0	1.40	0.18
NC_008716	Maracuja mosaic virus	6794	109.5	74.7	15.2	1.47	0.20
NC_008614	Cucumber mottle virus	6485	114.1	79.5	15.5	1.44	0.19
NC_004422	Youcai mosaic virus	6303	115.9	90.7	10.9	1.28	0.12
NC_003878	Zucchini green mottle mosaic virus	6513	104.5	64.4	17.7	1.62	0.27
NC_003610	Kyuri green mottle mosaic virus	6514	105.0	75.5	13.2	1.39	0.17
NC_001728	Odontoglossum ringspot virus	6618	100.9	71.7	13.3	1.41	0.19
NC_008365	Streptocarpus flower break virus	6279	116.0	82.2	14.9	1.41	0.18
NC_008295	Penstemon ringspot virus	6179	111.7	75.0	16.2	1.49	0.22
NC_004106	Paprika mild mottle virus	6524	98.9	70.9	12.3	1.39	0.17
NC_003852	Obuda pepper virus	6507	101.2	72.5	12.6	1.40	0.17
NC_003355	Crucifer tobamovirus	6298	100.4	78.3	9.3	1.28	0.12
NC_002792	Ribgrass mosaic virus	6301	112.4	90.6	9.3	1.24	0.10
NC_002633	Cucumber fruit mottle mosaic virus	6562	95.8	71.1	10.8	1.35	0.15
NC_001873	Turnip vein-clearing virus	6311	115.2	79.1	16.2	1.46	0.20
NC_001367	Tobacco mosaic virus	6395	109.3	78.3	13.4	1.40	0.17
■ Togaviridae							
NC_006558	Getah virus	11597	232.5	182.1	22.6	1.28	0.12
NC_004162	Chikungunya virus	11826	238.4	180.6	26.6	1.32	0.15
NC_003900	Aura virus	11824	229.3	178.0	23.2	1.29	0.13
NC_003899	Eastern equine encephalitis virus	11675	228.4	175.2	24.7	1.30	0.14
NC_003417	Mayaro virus	11411	217.8	160.6	26.4	1.36	0.16
NC_003215	Semliki forest virus	11442	219.5	165.9	24.3	1.32	0.15
NC_001547	Sindbis virus	11703	230.2	176.5	24.3	1.30	0.14
NC_001545	Rubella virus	9755	197.0	150.4	21.3	1.31	0.14
NC_001512	O'nyong-nyong virus	11835	239.0	175.0	28.8	1.37	0.16
NC_001449	Venezuelan equine encephalitis virus	11444	214.3	167.5	21.2	1.28	0.13
NC_003908	Western equine encephalomyelitis virus	11484	233.3	178.9	25.4	1.30	0.14
NC_001786	Barmah Forest virus	11488	219.3	174.5	20.5	1.26	0.12
NC_003433	Sleeping disease virus	11900	237.6	178.0	27.5	1.33	0.15
NC_003930	Salmon pancreas disease virus	11919	234.7	173.5	28.2	1.35	0.16
■ Tymoviridae							
NC_001746	Kennedya yellow mosaic virus	6362	115.0	75.9	16.3	1.52	0.21
NC_009532	Okra mosaic virus	6223	108.5	81.4	11.8	1.33	0.15
NC_007609	Dulcamara mottle virus	6181	114.7	84.0	13.3	1.37	0.16
NC_004063	Turnip yellow mosaic virus	6318	123.6	96.3	11.6	1.28	0.12
NC_003634	Physalis mottle virus	6673	113.2	81.6	14.0	1.39	0.17
NC_002588	Chayote mosaic virus	6364	101.8	70.6	13.7	1.44	0.19
NC_001977	Erysimum latent virus	6035	112.6	89.4	9.8	1.26	0.11
NC_001513	Ononis yellow mosaic virus	6211	102.7	72.3	13.0	1.42	0.18
NC_001480	Eggplant mosaic virus	6331	113.7	84.1	12.7	1.35	0.15

Table S2: RNA lengths and branching statistics for the 11 families of viruses studied. Numbers of vertices for each sequence are averages calculated for ensembles of 1000 predicted secondary structures (see Methods). RNA sequences are accessible by searching for the NCBI ID at http://www.ncbi.nlm.nih.gov/genome.
